# An evaluation of implementation climate in inpatient maternity care: a cross-sectional survey study

**DOI:** 10.1080/14767058.2023.2185119

**Published:** 2023-12

**Authors:** Rebecca F. Hamm, Lisa D. Levine, Elizabeth Quigley, Rinad S. Beidas

**Affiliations:** a Department of Obstetrics & Gynecology, University of Pennsylvania Perelman School of Medicine, Philadelphia, PA, USA; b Leonard Davis Institute of Health Economics, Perelman School of Medicine, University of Pennsylvania, Philadelphia, PA, USA; c Department of Nursing, University of Pennsylvania Perelman School of Medicine, Philadelphia, PA, USA; d Department of Medical Social Sciences, Feinberg School of Medicine, Northwestern University, Chicago, IL, USA

**Keywords:** Evidence-based practices, implementation climate, implementation climate scale, maternity care

## Abstract

**Objective:**

Researchers in obstetrics and gynecology are continuously generating new evidence to inform clinical care delivery. Yet, much of this newly emerging evidence fails to be rapidly and effectively integrated into routine clinical practice. Implementation climate refers to clinicians’ perceptions of to what degree organizations support and reward use of an evidence-based practice (EBP) and is an important construct in the science of implementation in healthcare. Little is known about implementation climate for EBPs in maternity care. Thus, we aimed to (a) determine the reliability of the Implementation Climate Scale (ICS) for use in inpatient maternity care, (b) describe implementation climate in inpatient maternity care overall, and (c) compare individual perceptions of implementation climate between physician and nursing clinicians on these units.

**Study design:**

We performed a cross-sectional survey of clinicians in inpatient maternity units across 2 urban, academic hospitals in the northeastern United States in 2020. Clinicians completed the 18-question validated ICS [scored 0–4]. Scale reliability by role was evaluated using Cronbach’s α. Subscale and total scores were described overall and compared by physician versus nursing role using independent t-tests, as well as linear regression to control for confounders.

**Results:**

111 clinicians completed the survey (physicians = 65; nursing = 46). Physicians were less likely to identify as female (75.4% vs. 100.0%, *p* < .001), but were of similar age and years of experience as nursing clinicians. Reliability of the ICS was excellent, with Cronbach’s α of 0.91 and 0.86 among physicians and nursing clinicians, respectively. Scores were notably low for implementation climate in maternity care overall and for all subscales. ICS total scores were also higher among physicians as compared to nurses (2.18(±0.56) vs. 1.92(±0.50), *p* = .02), which remained significant in multivariable modeling (*p* = .02). Unadjusted subscale scores were higher among physicians in Recognition for EBP (2.68(±0.89) vs. 2.30(±0.86), *p* = .03) and Selection for EBP (2.24(±0.93) vs. 1.62(±1.04), *p* = .002). After adjustment for potential confounders, subscale scores for Focus on EBP (*p* = .04) and Selection for EBP (*p* = .002) were all higher among physicians.

**Conclusions:**

This study supports the ICS as a reliable scale for measuring implementation climate in the inpatient maternity care setting. Notably lower implementation climate scores across subscales and roles compared to other settings may underlie the vast evidence to practice gap in obstetrics. In order to successfully implement practices that reduce maternal morbidity, we may need to focus on building educational support and rewarding EBP utilization on labor and delivery units, with an emphasis on nursing clinicians.

## Introduction

In maternity care, peripartum morbidity and mortality remain a substantial threat worldwide [1,2]. There are significant, unacceptable disparities by race, socioeconomic status, and ethnicity in birth outcomes [3,4]. Evidence-based practices (EBPs) have been developed to improve obstetric outcomes and reduce disparities in care [5-7]. Yet, much of this newly emerging evidence fails to be rapidly and effectively integrated into routine clinical practice. The evidence to practice gap in maternity care remains wide [8,9]. The underlying causes of this gap are likely multifactorial, and remain poorly elucidated.

Prior work has demonstrated that a key determinant of effective implementation of EBPs in healthcare is implementation climate, or the extent to which intended users perceive that use of EBPs are expected, supported, and rewarded [10]. Leading theories posit that the stronger the implementation climate, the more consistently EBPs will be utilized in care at a given site [11], and empirical work has begun to substantiate this [12]. The Implementation Climate Scale (ICS) was developed as a brief, pragmatic measure of six dimensions, or subscales, of implementation climate [13]. The ICS has been validated in several settings, including child welfare services, substance use disorders treatments, and inpatient nursing units [14-16]. The climate around EBP implementation in maternity care, potentially impacting the research to practice gap in obstetrics, has not specifically been studied.

In addition, while the ICS has been validated in nursing literature [16], there has been little work evaluating differences in perceived implementation climate by role in a health care setting, particularly comparing physicians and nurses. While implementation climate is meant to be assessed at the organizational level and not at the level of the individual [11], perceptions of implementation climate may differ by role within the institution [17,18]. In theory, support or rewards for use of EBPs could easily differ among the often distinct hierarchical structures of physicians and nursing, with varied perceptions on control over EBP implementation by role.

Thus, we aimed to (a) determine the reliability of the Implementation Climate Scale (ICS) for use in inpatient maternity care, (b) describe implementation climate in inpatient maternity care overall, and (c) compare individual perceptions of implementation climate between physician and nursing clinicians on these units. We anticipated that this analysis would reveal critical targets in the implementation climate in inpatient maternity care. Such targets could help close the research to practice gap in obstetrics.

## Materials and methods

We performed a cross-sectional survey of clinicians in inpatient maternity units across two urban, academic hospitals in the University of Pennsylvania Health System in Philadelphia, PA from November 2020 to December 2020. These sites are housed under the same administrative umbrella and perform a combined nearly 10,000 deliveries per year. Site #1 is an urban tertiary care hospital with a clinician mix including resident physicians under the supervision of maternal-fetal medicine (MFM) or academic generalists, private practice OB/GYNs, family practitioners, nurses, and nurse-midwives. Site #2’s clinician mix is predominantly private obstetricians, nurses, and nurse-midwives. Both sites are Magnet^®^ accredited and have robust hospital wide quality improvement initiatives. The project was approved by the University of Pennsylvania Institutional Review Board and written informed consent was obtained from all participants. CROSS guidelines for reporting of survey studies were utilized in this manuscript [19].

Clinicians caring for patients in maternity care settings at these two sites, including physicians, nurses, and nurse-midwives were invited to participate anonymously *via* institutional email listserves. There were no exclusion criteria.

The survey included an adapted form of the Implementation Climate Scale [13] where the word “team/agency” from the original scale was replaced with “my obstetric inpatient unit.” The ICS, composed of 18 items (survey questions), measures 6 dimensions (subscales) for EBP implementation. Each subscale is made up of 3 of the items. The subscales include: Focus on EBP, Educational support for EBP, Recognition for EBP, Rewards for EBP, Selection for EBP, and Selection for Openness. Examples of items targeted to our EBP include: “One of my obstetric inpatient unit’s main goals is to use evidence-based practices effectively” and “My obstetric inpatient unit provides conferences, workshops, or seminars focusing on evidence-based practices.” Response format is a 4-point Likert scale with higher scores indicating greater value. Total scores are averaged across all 18 items, thus range from 0 to 4. Scores for each of the 6 subscales are averaged among the 3 items encompassing that subscale, again ranging from 0 to 4. Demographic data was also elicited from participants.

In analysis, participants were grouped by role, into physician and nursing clinicians. Nursing clinicians included registered nurses, certified nurse midwives, and nurse-practitioners. Physician clinicians included both attending level and resident trainee physicians from obstetrics/gynecology, attending level and fellow trainee physicians from maternal fetal medicine, and attending level and resident trainee physicians from family medicine. Demographic data of participants was compared by clinician role using chi-square and Fisher’s exact test for categorical variables, and Student’s t-test and Wilcoxon rank sum for continuous variables, where appropriate.

Internal reliability of the scale in the inpatient maternity care setting was determined overall by role, as well as for each subscale dimension using Cronbach’s α. Thus, individual perceptions of implementation climate were evaluated for this analysis. As a result, while ICS scores from the 2 sites were grouped, site was included as a possible confounder in all adjusted models.

ICS total and individual dimension scores were compared by role using Student’s t-test. Linear regression was used to control for possible confounders of the relationship between clinician role and ICS score. In addition to site, we evaluated demographic and clinical characteristics associated on bivariate tests (*p* < .20) with the exposure (clinician role) as well as the outcome of interest (ICS score) as potential covariates. After backwards stepwise elimination, covariates with *p* < .10 in multivariable modeling were retained in final models. Thus, site, gender identity and years of experience were evaluated as potential confounders in all models. Statistical analyses were performed with Stata 15 (StataCorp, College Station, TX). All tests were 2-tailed, and *p*-values <.05 were considered statistically significant.

While sample size was based on the number of participants responding the survey, a sample size of 111 participants at a physician-nursing ratio of 1.2 had more than 80% power to demonstrate a 15% lower total ICS score for nursing clinicians at an alpha of 0.05, assuming a physician mean total ICS of 2.2 ± 0.5.

## Results

There were 111 participants who completed the ICS for an inpatient maternity care setting. Among participants, 65 (58.6%) were physician clinicians and 46 (41.4%) were nursing clinicians. Physician clinicians were 38.5% resident and fellow trainees (*n* = 25) and 61.5% (*n* = 40) attending physicians. Physicians were 61.5% (*n* = 40) general obstetricians/gynecologists, 26.2% (*n* = 17) maternal-fetal medicine physicians, and 12.3% (*n* = 8) family medicine physicians. Among nursing clinicians, 40 (87.0%) were RNs, 5 (10.9%) certified nurse-midwives, and 1 (2.2%) certified nurse practitioner.

When comparing demographics of physician versus nursing clinicians, physicians were less likely to identify as female (75.4% vs. 100.0%, *p* < .001) with no significant differences in age, practice site, or years in practice (Table 1).

Reliability of the ICS overall was excellent, with Cronbach’s α of 0.89 overall, and 0.91 and 0.86 among physicians and nursing clinicians, respectively. Each of the 6 subscale reliabilities were also acceptable, with Cronbach’s α of 0.75 or higher among both groups of clinicians (Table 2).

Table 3 reports overall ICS and subscale scores for the cohort as compared to other work utilizing the ICS. When comparing ICS scores between this work and prior studies in other organizations and practice types, total scores were lower than 2 of 4 studies shown. Total ICS score in maternity care was most similar to the study performed in substance use disorder treatment organizations. In addition, scores were lowest in the maternity care setting for Rewards for EBP, Selection for EBP, and Selection for Openness as compared with other work using the ICS.

In comparing ICS scores by clinical role (Table 4), ICS total scores were higher among physician as compared to nursing clinicians (2.18 ± 0.56 vs. 1.92 ± 0.50), *p* = .02), which remained significant in multivariable modeling (*p* = .03). Unadjusted subscale scores were higher among physicians in Recognition for EBP (2.68 ± 0.89) vs. 2.30 ± 0.86), *p* = .03) and Selection for EBP (2.24 ± 0.93) vs. 1.62 ± 1.04), *p* = .002). After adjustment for potential confounders, subscale scores for Focus on EBP (*p* = .04), and Selection for EBP (*p* = .002) were higher among physicians. There were no significant differences in Education Support for EBP, Recognition for EBP, Rewards for EBP, or Selection for Openness among physician and nursing clinicians.

## Conclusions

First, this study supports the ICS as a reliable scale for measuring individual perceptions of implementation climate in the inpatient maternity care setting. A scale for measuring climate around EBP implementation in maternity care may help organizations identify areas for focus and improvement to support implementation efforts.

Next, total and several subscale scores for implementation climate were notably low in maternity care compared to some other settings, with particularly low scores in Rewards for EBPs. This finding is particularly meaningful and surprising in the context of the urgent need for inpatient maternity units to implement evidence that can improve maternal morbidity and mortality. It is not unreasonable to consider that poor climate around implementation may be a substantial factor in the evidence-to-practice gap in obstetrics. However, Rewards for EBPs has also been identified as the lowest scoring dimension within the ICS in many of the prior populations for which the tool has been validated, such as med-surg nursing units and substance use disorder treatment centers [13-16]. While there is likely difficulty with providing rewards for EBPs, such as financial incentives, due to resource limitations, these strategies have demonstrated success in improving implementation endeavors [20-22] and may represent a critical area for focus across settings for quality improvement efforts.

Finally, there are differences in perception of implementation climate by clinician role within this setting that may be generalizable to other settings as well, thus offering an important area for future research within the field. Such results bring up the question whether implementation climate should be aggregated by role in future work within organizations. Nursing clinicians have lower scores overall, as well as in several ICS subscales. While it is often assumed that implementation climate is an organizational measure, it is possible that these differences by role can be explained by the often parallel hierarchies of physician and nursing leadership. It is possible that because of these separate structures, clinicians might perceive concepts like “Focus on EBP” and “Support for EBP” differently. Lower scores in these domains for nursing clinicians may also reflect the often unrecognized contribution of nursing to clinical care and the resulting impact on ownership of EBP implementation. The differences in perception of implementation climate by role may reveal an untapped area for focus to improve implementation climate in maternity care settings. Organizations should assess what resources for EBP implementation are available, as well as promote a climate of recognition for utilizing EBPs, specifically among nursing clinicians. Tailored strategies to promote implementation climate in nursing may need to be separate, parallel, or interwoven with those in place for physicians depending on the organizational structure.

Strengths of this work include the utilization of a validated measure for implementation climate, and its evaluation in a context with a significant evidence to practice gap. Study of determinants of implementation success in maternity care are of utmost importance to promote reduction in maternal morbidity and mortality. Limitations of the work includes the survey distribution to listserves of eligible clinicians, of which is it difficult to determine an accurate number who received the email (i.e. denominator), which is likely close to 350. Demographic data was not obtained on those who did not complete the survey, and the self-selecting population who completed the ICS may have been biased in their perceptions around implementation. Next, our sample is limited to 2 urban, academic sites within one geographic location and hospital system, possibly limiting generalizability, particularly around measures of reliability. However, results of our work validating the ICS are overall similar to the other populations and practice models in which it has been studied. Advanced practice nursing clinicians were grouped here in the nursing cohort, which reflects the organizational structure of our institution, but may not accurately reflect organizational structure elsewhere; however, these clinicians were only a small subset of our population. Furthermore, in this study, we have explored individual perceptions of intra-organizational factors rather than aggregated organizational level constructs. ICS scores differed significantly among clinicians across our 2 sites; however, these differences were controlled for in all multivariable analyses comparing individual perceptions by clinician role.

Our work demonstrates that implementation climate is lacking in maternity care, which may underlie why effective practices to reduce maternal morbidity and mortality are not widespread. Importantly, others have evaluated effective interventions for improving and sustaining improvement in implementation [23]. The leadership and organizational change for implementation (LOCI) intervention includes leadership training for workgroup leaders, ongoing implementation leadership coaching, 360° assessments, and strategic planning with top and middle management regarding how they can support workgroup leaders in developing a positive EBP implementation climate. Future directions might specifically focus on taking LOCI to the maternity care setting with a focus on building educational support, rewarding EBP utilization, and emphasizing nursing.

This study supports the Implementation Climate Scale as a reliable tool in the inpatient maternity care setting, for both physician and nursing clinicians. In this setting, nursing clinicians reported lower implementation climate scores than physician clinicians, revealing what may be an important opportunity to strengthen organizational capacity for EBP implementation. Notably, lower implementation climate scores across subscales and roles compared to other settings may underlie the evidence to practice gap in obstetrics. These results should encourage focus, both from the research and practice sides, on improving implementation climate in obstetrics, a determinant of implementation success.

## Figures and Tables

**Table 1.** Demographics of survey participants by clinician role.

		Physician Clinicians (*n* = 65) *n* (%)	Nursing Clinicians (*n* = 46) *n* (%)	*p*-value
Age (years)^a^		36 [31–43]	37 [32–43]	.34
Site	1	36 (55.4)	22 (47.8)	.43
	2	29 (44.6)	24 (52.2)	
Gender identity	Female	49 (75.4)	46 (100.0)	<.001
	Male	15 (23.1)	0 (0)	
	Other/Prefer not to respond	1 (1.5)	0 (0)	
Years in practice	0–2	15 (23.1)	4 (8.7)	.10
	3–5	16 (24.6)	7 (15.2)	
	6–10	9 (13.9)	13 (28.3)	
	11–20	16 (24.6)	13 (28.3)	
	>20	9 (13.9)	9 (20.0)	

a Median [IQR].

**Table 2.** Reliability of Implementation Climate Scale (ICS) overall and by clinician role.

	Cronbach’s α		
	Overall *N* = 111	Physician *N* = 65	Nursing *N* = 46
Total ICS Score	0.89	0.91	0.86
Subscale 1: Focus on EBP	0.88	0.89	0.85
Subscale 2: Educational Support for EBP	0.87	0.91	0.75
Subscale 3: Recognition for EBP	0.82	0.79	0.84
Subscale 4: Rewards for EBP	0.89	0.93	0.85
Subscale 5: Selection for EBP	0.89	0.87	0.90
Subscale 6: Selection for Openness	0.94	0.95	0.92

**Table 3.** Implementation Climate Scale (ICS) scores in maternity care as compared to other work utilizing the ICS.

Setting	Inpatient maternity care	Florida Med-surg nursing	Substance use disorder treatment organizations	Child welfare organizations	Mental health agencies
Reference (*n*)	This work *N* = 111	Ehrhart et al. 2021 *N* = 301	Ehrhart et al. 2019 *N* = 326	Ehrhart et al. 2016 *N* = 215	Ehrhart et al. 2014 *N* = 630
Total ICS Score	2.07 (0.55)	2.51 (0.85)	2.04 (0.66)	2.23 (0.66)	1.93 (0.73)
Subscale 1: Focus on EBP	2.98 (0.76)	3.09 (0.80)	2.63 (0.88)	2.96 (0.90)	2.28 (1.04)
Subscale 2: Educational Support for EBP	2.19 (0.96)	2.61 (1.04)	2.25 (1.03)	2.62 (1.01)	2.00 (1.09)
Subscale 3: Recognition for EBP	2.51 (0.89)	2.60 (1.13)	1.86 (0.97)	1.89 (1.00)	1.68 (1.10)
Subscale 4: Rewards for EBP	0.29 (0.59)	1.73 (1.26)	0.64 (0.88)	0.82 (0.97)	0.72 (0.93)
Subscale 5: Selection for EBP	1.98 (1.02)	2.14 (1.17)	2.23 (0.97)	2.30 (1.00)	2.09 (1.00)
Subscale 6: Selection for Openness	2.35 (0.84)	2.85 (0.97)	2.63 (0.92)	2.83 (0.87)	2.79 (0.91)

**Table 4.** Implementation Climate Scale (ICS) scores overall and subscale as compared among physician and nursing clinicians, both unadjusted and adjusted.

	Score [scale 0–4] Mean (SD)		Physician compared to nursing *p*-value	
	Physician	Nursing	Unadjusted	Adjusted
Total ICS Score	2.18 (0.56)	1.92 (0.50)	.02	.03^a^
Subscale 1: Focus on EBP	3.10 (0.80)	2.81 (0.68)	.052	.04^b^
Subscale 2: Educational Support for EBP	2.33 (1.05)	1.98 (0.76)	.055	.08^a^
Subscale 3: Recognition for EBP	2.68 (0.89)	2.30 (0.86)	.03	.050^a^
Subscale 4: Rewards for EBP	0.30 (0.60)	0.29 (0.58)	.95	.95^c^
Subscale 5: Selection for EBP	2.24 (0.93)	1.62 (1.04)	.002	.002^c^
Subscale 6: Selection for Openness	2.28 (0.86)	2.46 (0.82)	.27	.27^c^

a Controlling for site.

b Controlling for site and years of experience.

c No covariates retained in the model.
